# “The role as a champion is to not only monitor but to speak out and to educate”: the contradictory roles of hand hygiene champions

**DOI:** 10.1186/s13012-019-0943-x

**Published:** 2019-12-23

**Authors:** Cassie Cunningham Goedken, Daniel J. Livorsi, Michael Sauder, Mark W. Vander Weg, Emily E. Chasco, Nai-Chung Chang, Eli Perencevich, Heather Schacht Reisinger

**Affiliations:** 1 Center for Access & Delivery Research and Evaluation, Iowa City VA Health Care System, 601 Hwy 6 West, Iowa City, 52246 USA; 2 Department of Internal Medicine, Carver College of Medicine, University of Iowa, 375 Newton Rd, Iowa City, IA 52242 USA; 3 Department of Sociology, University of Iowa, 140 Seashore Hall West, Iowa City, IA 52242 USA; 4 Department of Psychological and Brain Sciences, University of Iowa, W311 Seashore Hall, Iowa City, IA 52242-1407 USA; 5 University of Utah School of Medicine, 30 N 1900 E, Salt Lake City, UT 84132 USA

**Keywords:** Hand hygiene, Champion, Implementation science, Implementation strategy, Infection prevention

## Abstract

**Background:**

Implementation science experts define champions as “supporting, marketing, and driving through an implementation, overcoming indifference or resistance that the intervention may provoke in an organization.” Many hospitals use designated clinical champions—often called “hand hygiene (HH) champions”—typically to improve hand hygiene compliance. We conducted an ethnographic examination of how infection control teams in the Veterans Health Administration (VHA) use the term “HH champion” and how they define the role.

**Methods:**

An ethnographic study was conducted with infection control teams and frontline staff directly involved with hand hygiene across 10 geographically dispersed VHA facilities in the USA. Individual and group semi-structured interviews were conducted with hospital epidemiologists, infection preventionists, multi-drug-resistant organism (MDRO) program coordinators, and quality improvement specialists and frontline staff from June 2014 to September 2017. The team coded the transcripts using thematic content analysis content based on a codebook composed of inductive and deductive themes.

**Results:**

A total of 173 healthcare workers participated in interviews from the 10 VHA facilities. All hand hygiene programs at each facility used the term HH champion to define a core element of their hand hygiene programs. While most described the role of HH champions as providing peer-to-peer coaching, delivering formal and informal education, and promoting hand hygiene, a majority also included hand hygiene surveillance. This conflation of implementation strategies led to contradictory responsibilities for HH champions. Participants described additional barriers to the role of HH champions, including competing priorities, staffing hierarchies, and turnover in the role.

**Conclusions:**

Healthcare systems should consider narrowly defining the role of the HH champion as a dedicated individual whose mission is to overcome resistance and improve hand hygiene compliance—and differentiate it from the role of a “compliance auditor.” Returning to the traditional application of the implementation strategy may lead to overall improvements in hand hygiene and reduction of the transmission of healthcare-acquired infections.

## Background

Hand hygiene is widely considered the most effective method of preventing healthcare-acquired infections (HAIs) [1, 2]. However, hand hygiene compliance remains persistently low [1–4]. In the USA, the Joint Commission’s 2019 National Patient Safety Goal challenges hospitals to lower the risk of HAI by utilizing current Centers for Disease Control and Prevention [5] or World Health Organization hand hygiene guidelines [2, 6]. In addition, it recommends that hospitals set their own goals for hand hygiene compliance and work toward improving compliance toward those goals [7]. One approach many hospitals use to improve hand hygiene compliance is the recruitment of clinical champions—often called “hand hygiene (HH) champions.”

It is well accepted that champions are important in implementation and quality improvement projects [8]. Champions have been defined within the field of implementation science as “individual(s) who dedicate themselves to supporting, marketing, and driving through an implementation, overcoming indifference or resistance that the intervention may provoke in an organization” ([9], p. 9). The concept of a champion to influence change has existed for several decades [10–12]. Although the definition has evolved, many key aspects remain the same; namely, champions are dedicated individuals who attempt to influence and elicit change. Champions are often referred to by a variety of different titles, such as opinion leaders, sponsors, internal entrepreneurs, and change agents [13, 14].

Versions of champions have also been used successfully to improve hand hygiene compliance. Hand washing champions were identified as playing a key role in improving physician hand hygiene compliance at a large, urban hospital in the Midwestern USA [15]. A new resident-trained hand washing champion was identified each day. They provided immediate feedback to inpatient general pediatric teams regarding hand hygiene performance and verbal reminders that were associated with a sustained increase in hand hygiene compliance [15]. Similarly, Saint and colleagues [16] increased healthcare worker (HCW) hand hygiene compliance across five units in Tuscany, Italy, with a multimodal intervention that included identifying champions on each hospital unit through the presence of green buttons on their clothing promoting hand hygiene, as well as modeling proper hand hygiene behavior. Further, Patel et al. [17] reported hand hygiene compliance improvements before and after patient contact by employing hand hygiene champions to facilitate a variety of intervention activities, including HCW education and providing feedback to units based on audits conducted by an independent infection control team at a hospital in Cape Town, South Africa.

Despite the success of champions used in specific interventions, little is known about whether and how healthcare systems have employed the implementation strategy of clinical champions to promote hand hygiene. As the field of implementation science matures, it is critical to examine how implementation strategies are being used and defined in real-world settings. While it is difficult to find a single, well-accepted definition of ethnography [18, 19], most would agree “its emphasis is on the description and analysis of “the everyday”—routine behaviors in their natural settings ([20], p.326).” The goal of this study was to conduct ethnographic research of hand hygiene programs at 10 Veterans Health Administration (VHA) hospitals to understand key components and “everyday” practices of the programs prior to conducting a cluster-randomized control trial of specific hand hygiene interventions [21]. Ethnographic examination of program components as they are discussed and used by healthcare workers illuminates the gaps between conceptualizations defined by experts and real-world applications. One of the key practices identified at each of the facilities was hand hygiene champions. In this paper, we examine the real-world use of the term “champion,” including whether the term was used in these programs, how the term was defined, and what the role of HH champions entailed when used to promote hand hygiene compliance. Conducting this research allowed us to compare the use of the term among the participating Veterans Affairs (VA) hospitals with its application in the literature.

## Methods

**Fig. 1:**
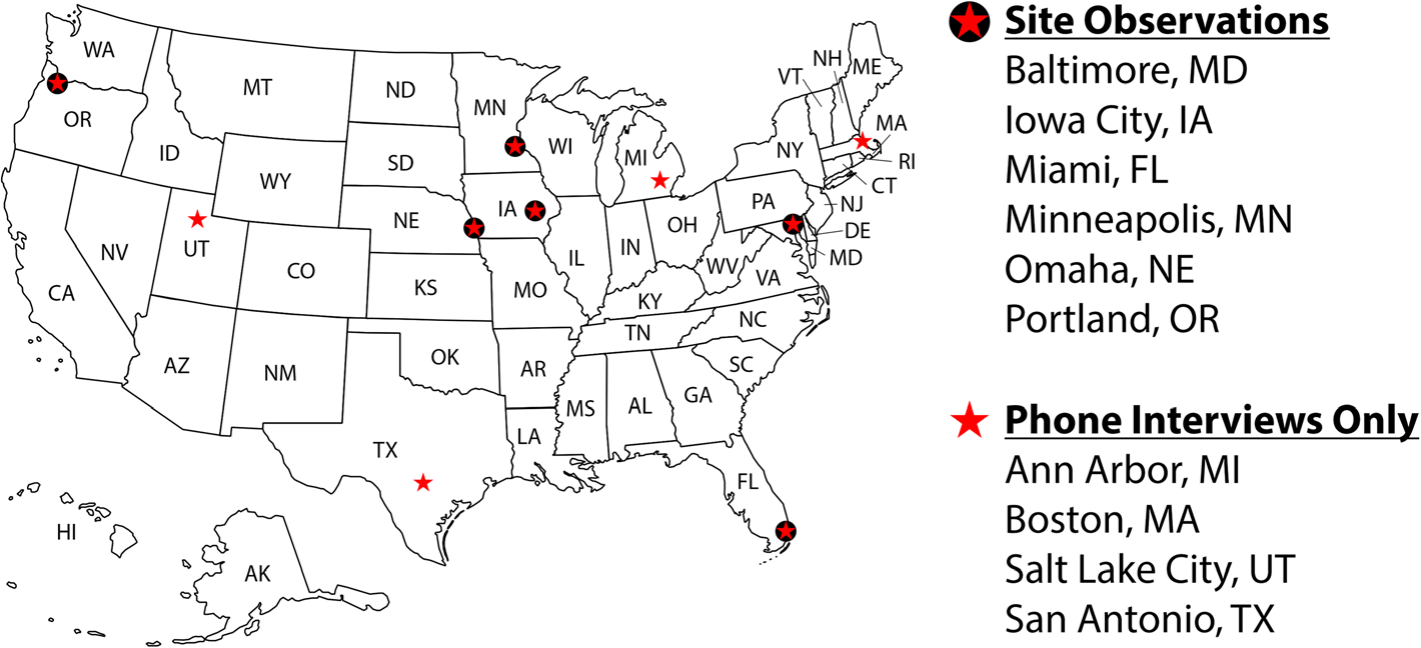
Qualitative evaluation

As part of a multicenter cluster-randomized trial testing three hand hygiene improvement interventions, an ethnographic study was conducted with infection control teams and frontline staff directly involved with hand hygiene across 10 geographically dispersed Department of Veterans Affairs Healthcare Systems (VAHCSs) in the USA. We conducted site visits at six facilities, which included observations of infection control practices, semi-structured interviews with staff directly involved with hand hygiene (hospital epidemiologists, infection preventionists, multi-drug-resistant organism (MDRO) program coordinators, and quality and patient safety staff), and focus groups with frontline staff (two different units, one day shift and one night shift). Staff directly involved with hand hygiene participated in semi-structured phone interviews at four additional VAHCS facilities (Fig. 1). Data were collected at two different time points. The first round of data collection (Time 1 [T1]) occurred between June 2014 and March 2015, and the second round (Time 2 [T2]) occurred between January 2017 and September 2017. T1 data collection was conducted prior to implementing hand hygiene improvement interventions for the cluster-randomized trial. For T2 data collection, we conducted site visits at four of the original six hospitals since two hospitals were unable to implement the study interventions due to lack of personnel.

An ethnographic approach was chosen for both methodological and epistemological reasons [22]. From the breath of methods within the ethnographic methodology [23, 24], we chose to conduct semi-structured individual and group interviews and observations at six sites and supplement the data with phone interviews from an additional four sites. We also collected organizational documents (e.g., hand hygiene protocols, data collection forms) from all 10 sites. For this paper, we focus on the semi-structured interview data due to the emphasis on how they defined “hand hygiene champions” and the need to focus on the in vivo language used by the participants. We also report on our review of organizational documents and observations to provide context on the organizational structure of the hospitals’ hand hygiene programs.

The semi-structured interview guides were developed by the interdisciplinary research team who had substantial expertise in hospital epidemiology and infection prevention. The interview guides were iteratively revised as new data was collected and based on feedback from the participants. Interview guide questions included questions to elicit past and current strategies used for promoting hand hygiene compliance, descriptions of hand hygiene surveillance practices, and how roles and responsibilities related to hand hygiene were structured at the facility level. The focus group guide was developed in the same way and included questions about frontline staff’s (e.g., nurses, physicians, unit clerks, and environmental service workers) knowledge and perceptions of strategies to promote and monitor hand hygiene compliance on their unit and in their facility more broadly. We also collected data on hospital staff perceptions of the three bundled interventions, but those data were not included in this analysis.

Interviews were audio recorded and transcribed by trained transcribers. Transcriptions were reviewed against original recordings by the interviewers. All transcripts were imported into MAXQDA qualitative data management software [25]. The study was approved by the VA Central Institutional Review Board and Research and Development Committee at the Iowa City VAHCS, and informed consent was reviewed with all participants.

### Analysis

We conducted a thematic content analysis [26, 27] with an interdisciplinary team that included ethnographically trained social scientists from anthropology and sociology, a health psychologist with experience in infection prevention, and a hospital epidemiologist. Data from T1 of the collection were analyzed across four phases. First, three transcripts were reviewed by the analysis team, and a codebook was developed based upon a priori research questions and emergent content [28]. During the first phase, the transcripts were coded using parallel processes. Forty-nine percent of transcripts were coded by consensus at biweekly meetings among the analysis team. All coders participated in the biweekly meetings. Two coders from the analysis team coded the remaining transcripts (51.2%) during this same period. Each coder first coded independently and then met to reach consensus and enter the coding in MAXQDA. This process of continuous dialogue increases the validity and reliability of the coding process by refining the content boundaries of codes and improving coding consistency.

In the second phase of analysis with T1 data, we subcoded the two most frequently applied codes “hand hygiene intervention strategies” and “hand hygiene monitoring.” “HH champion” was a subcode that cut across both high-frequency codes. We applied the subcode to segments of text in which “HH champion” was used verbatim (in vivo). We also noted segments of text that were conceptually similar, but in which the term “HH champion” was not used. Conceptually, “HH champion” included references to individuals responsible for surveillance, peer-to-peer coaching, formal and informal education, and general promotion of hand hygiene on a unit or within their own clinical group. We subcoded all text segments under these conceptually related categories as “HH champion.”

In phase three of the analysis, we used the hierarchical codebook (codes and subcodes) developed with T1 data to code all transcripts from the T2. Thirty-five percent of transcripts were coded via group consensus, and 65% were coded by at least two coders.

In the final phase, we tested the “HH champion” subcode by conducting a lexical search of all occurrences of “champion” (or champions) in the transcripts and compared the lexical search to all “HH champion”–coded segments.

## Results

A total of 38 individuals responsible for or involved with hand hygiene programs participated in semi-structured interviews, while 70 frontline staff participated in focus groups in the T1 interviews. In T2 interviews, 32 individuals responsible for or involved with hand hygiene programs participated in semi-structured interviews, and 33 frontline staff participated in focus groups.

Descriptive characteristics of staff are included in Table 1 (T1) and Table 2 (T2). Infection preventionists (T1, 14%; T2, 19%) composed the largest proportion of staff, with the remainder divided among hospital epidemiologists (T1, 9%; T2, 12%), MDRO program coordinators (T1, 7%; T2, 6%), and others (e.g., quality and patient safety) (T1, 7%; T2, 12%). The largest proportion of the frontline staff sample was composed of nurses (T1, 64%; T2, 46%).

**Table 1:** First round data collection (T1) participant characteristics at 10 VAHCs hospitals

Individuals responsible for HH characteristic	Total participants (*N* = 38)
Mean age (SD)	49.91 (9.23)
Mean tears at VA (SD)	13.85 (9.96)
Occupation	
Hospital epidemiologist	10
Infection preventionist	15
MDRO PC	7
Others (e.g., quality, patient safety)	6
Frontline staff characteristic	Total participants (*N* = 70)
Mean age (SD)	41.66 (11.2)
Mean years at VA (SD)	8.19 (9.07)
Occupation	
Nursing	53
Medial	3
EMS	3
Admin	3
Other	7

*MDRO PC*, multi-drug-resistant organism prevention program coordinator

**Table 2:** Second round data collection (T2) participant characteristics at 8 VAHCS hospitals

Individuals responsible for HH characteristic	Total participants (*N* = 32)
Mean age (SD)	47.31 (9.84)
Mean years at VA (SD)	11.93 (9.31)
Occupation	
Hospital epidemiologist	8
Infection preventionist	12
MDRO PC	4
Others (e.g., quality, patient safety)	8
Frontline staff characteristic	Total participants (*N* = 33)
Mean age (SD)	38.12 (11.84)
Mean years at VA (SD)	5.08 (6.00)
Occupation	
Nursing	30
EMS	2
Other	1

*MDRO PC*, multi-drug-resistant organism prevention program coordinator

### The concept of “HH champions”

Participants often referred to the concept of a “HH champion” to describe a range of roles, including monitoring hand hygiene compliance, informal and formal HCW education, and general promotion of hand hygiene. Of the 233 “HH champion”–coded segments, the lexical search showed 49.8% (*n* = 116) of participants specifically use the term “champion” and in all but one interview the participant used the term champion before the interviewer (i.e., without prompting by the interviewer). In our analysis, we found all 10 sites used both the concept of HH champions and the specific term.

### Organizational structure of hand hygiene programs

Based on analysis of the organizational documents (*n* = 10 sites) and observational field notes (*n* = 6 sites) and supplemented by interviews with hand hygiene program staff (*n* = 38), the organizational structure of hand hygiene programs varied across all 10 hospitals. Hand hygiene was managed by Infection Control at six hospitals, whereas in the remaining four facilities it fell under the umbrella of Quality and Patient Safety. Despite the difference in oversight, infection control teams at all 10 sites were responsible for reporting hand hygiene compliance rates to hospital leadership. Three of the six observed sites displayed hand hygiene compliance data on at least one unit of the hospital. All hand hygiene programs reported using HCWs to monitor the hand hygiene compliance of their fellow HCWs on their units. This is similar to findings from the 2012 survey of VHA facilities in which we found 98.6% of facilities used direct observation to monitor hand hygiene [29]. In addition to monitoring hand hygiene compliance, some were also responsible for entering observations into an electronic system for reporting. Many described this role as “HH champion”; however, the responsibilities of HH champions varied and often included providing peer-to-peer coaching, delivering informal education, and general promotion of hand hygiene, in addition to their surveillance role. Below, we present the analysis of the elements that emerged from the ethnographic data to define the role of HH champions as it played out in real-world clinical practice.

### HH champion: surveillance

The subtheme “surveillance” focused on the responsibility of collecting hand hygiene compliance data. Within this subtheme, participants used terms such as surveillance, monitoring, and observation and discussed whether or not the collection of compliance data was covert.

Many sites reported utilizing HH champions to conduct monitoring of hand hygiene compliance. This surveillance typically came from an individual chosen by the unit’s nurse manager, although sometimes the individual would volunteer for the role:

*… usually each area will identify a person to, you know, be a--, do their hand hygiene monitors and/or delegate the hand hygiene monitors, or just kinda be the champion for that area. [program analyst, site 6, T2]*

Some hospitals had covert observers, while other facilities wanted the observers to be known and able to provide immediate feedback. As one staff member in charge of a facility’s hand hygiene program noted:

*We used to call them secret shoppers. I prefer to call them hand hygiene champions, like people that are championing, that are collecting observations. Some people know who they are, some people don’t. They just know that there’s somebody watching them in their area, which I think is, is always good when you know people are out there watching you. [frontline staff, site 8, T2]*

Others intentionally chose not to have champions conduct observations covertly:

*We went out and we did teaching and the observers, they were no longer secret. They were observers and they were proactive, going to the person and let them know--, (I was) observing hand hygiene and, “I noticed that you came out. We would like you to have your hands wash [ed] or use hand sanitizer in order to prevent an (infection).” [quality/patient safety lead, site 9, T1]*

In these examples, surveillance is a part of the HH champion role, but informal education or coaching was also part of the responsibility at some sites.

### HH champion: formal and informal education

The subtheme “formal and informal education” refers to another responsibility held by the HH champions. Specifically, this theme describes the different styles/ways in which HH champions must educate fellow HCWs on hand hygiene practices, which may include encouragement/coaching, reminders, and informing/teaching. Although participants indicated that hand hygiene education was typically done both formally and informally, HH champions were generally tasked with informal education and peer-to-peer coaching.

*We (the infection control team) always encourage them [HH champions] to speak up. So if it is one of like our nurse observers, we would—, if they want to speak up, we don’t want them to come out themselves as somebody that may be doing observations, but if they could ( … ) try to encourage each other to do the right thing. [infection preventionist, site 8, T2]*

Having staff on the unit champion hand hygiene was considered desirable from an infection control standpoint. The belief was as follows: If staff are involved in hand hygiene by reminding and educating one another, this may help shift the culture on the unit and improve hand hygiene compliance by raising staff awareness.

*This (being the HH champion on a unit) is a constant reminder, the fact that they (HH champion) are doing this ( … ) it’s a reminder for them (HH champion) to participate in hand hygiene and to be well aware of people who don’t, you know. [infection preventionist, site 9, T1]*

*(A HH champion is) somebody who works in the area, who people know, who can really, you know, engage people to change their practice. [program analyst, site 6, T2]*

One nurse elaborated on this idea of culture change and explained how the observers on the units provided education to staff through direct feedback, which she believed could lead to making hand hygiene compliance routine.

*When we have people as champions they can change the culture of the floor. We definitely encourage them to say something. I’m constantly saying, “This is your floor; you protect that patient and you know if you see something not right you tell someone. You tell them.” You know this is a part of their role as a champion is to not only monitor but to speak out and to educate.” [infection preventionist, site 5, T1]*

### HH champion: barriers

“Barriers” is a subtheme we used to capture the many challenges associated with being the HH champion. Challenges ranged from difficulties navigating the hospital hierarchy to sufficient time for their HH champion responsibilities. We found unique barriers associated with the roles HH champions held. These barriers existed when HH champions were solely acting in the auditing or surveillance role, but they also proved to be a challenge when HH champions were tasked with the combined roles of auditors and educators.

As noted, having staff monitor hand hygiene on their own unit has its advantages*,* such as being able to provide education and immediate feedback to *peers—“their role as a champion is to not only monitor but to speak out and to educate.” [infection preventionist, site 5, T1].* However, this combined role also has its disadvantages. These disadvantages reflect the contradictory roles of the HH champion: the responsibility to accurately monitor and report the behavior on the unit for purposes of quality improvement and the responsibility to promote hand hygiene and educate peers.

*I: Okay. Um, are they secret champions or—?*

*3113: Um, some are and some aren’t. So it’s all with preference. I DO like to tell them to be known as a hand hygiene champion for their unit or their area. I try to tell them this, “If you’re observing someone and they’re consistently non-compliant and you don’t say anything, then yeah, you’re getting decent data, but you’re not—, you’re not acting on the cause.” We’re trying to prevent infections by increasing awareness, so I do try to push that they be known to their unit as a champion, if you’re around, people will do hand hygiene. [infection preventionist, site 5, T2]*

This same infection preventionist went on to talk about the challenges regarding the responsibilities of hand hygiene compliance in the context of staff hierarchies, which is a recurring theme among the barriers described below:

*“This is your patient. You are responsible for this patient. You’re, you’re responsible in preventing infections, and if you’re doing hand hygiene and then, say, a physician walks in and doesn’t do hand hygiene, well, you know, there goes all your effort.”*

In addition, staff who are observing their peers report feeling conflicted about their dual role as colleague/peer observer. They do not want to get their fellow HCWs or their unit in trouble, and therefore may report only the “good” observations they observe.

*We basically have champions in all our units, they do hand hygiene observations. ( … ) The disadvantage of our program is that in some ways you have the fox guarding the hen house so ( … ) you may have more positive responses than really are.” [hospital epidemiologist, site 5, T1]*

Further, through interviews with frontline staff, we found some peers request special treatment if they know who in the unit is collecting the observations.

*( … ) they’ll (frontline staff) step away and be like, “don’t write me up,” out there you know you (HH champion) give people chances [frontline staff, site 9, T2]*

HH champions whose primary role was to provide education and promote good hand hygiene often discussed the difficulties of navigating boundaries and dealing with the hierarchy within their facilities.

*I think people who are afraid to say something to someone who is maybe higher ranking, and remind them although we keep saying it’s okay to remind people, it’s important that you do. I think the nurses are--, after a while they get tired of you know telling the next crop of residents coming in, you know, surgical residents that don’t wash their hands, don’t put on gloves when they handle dressings, you know, after years and years of that, they kind of wear out I think. [infection preventionist, site 3, T1]*

One nurse in charge of hand hygiene at her facility explained how staff often defer this responsibility onto the infection control team.

*Hand Hygiene Nurse: When staff have concerns with a physician not practicing, they call [name of infection preventionist].*

*Interviewer: Is there any reason why?*

*Hand Hygiene Nurse: I think just because she’s higher up the chain of hierarchy and staff nurses don’t always feel comfortable challenging a doc about their hand hygiene or infection control practices. [infection control nurse, site 1, T2]*

Time and sustainability are common barriers cited by HH champions who serve in the hand hygiene auditing and education roles. We found insufficient time to be a disadvantage of tasking staff on the unit as HH champions. Frontline staff’s primary job is patient care. Therefore, they may be too busy to fulfill the role of the HH champion, adequately monitor hand hygiene compliance, and provide hand hygiene education, as well as coach other HCWs about proper hand hygiene compliance.

*I mean we’re asking nursing personnel, you know, to give up whatever they give up. I don’t know if they give up anything but, you know, they’re in charge of doing patient care and we’re asking them to do hand hygiene. And I know it creates a problem for some of them.” [MDRO program coordinator, site 10, T1]*

In addition, sustainability is commonly cited as a barrier to having floor staff champion hand hygiene. HH champion turnover, staff shortages, and staff reassignment to different units to accommodate the ebb and flow of the patient census contribute to this. Further, when staff become too busy to be the champion, it becomes a rotating role, which can reduce continuity and diffuse responsibility.

### Counter example: division of responsibilities

Although all sites used the term HH champion, the responsibilities assigned to the role were not uniform. Of the ten sites, two specifically attempted to maintain the anonymity of hand hygiene observers, although they still called the role HH champions. Other sites took advantage of opportunities such as using summer student interns to audit the HH champions’ observations, but these roles were not labeled as champions. However, barriers exist in this model as well.

*… one of the work areas here, the person that was the champion in that work area was actually identified by the manager in the area to just briefly discuss their experience with the process and the person actually came up here (infection prevention team office space) and said, “I feel kind of—, I’ve been outed by the manager. How successful am I going to be anymore?” [infection preventionist, site 4, T1]*

In short, this HH champion felt being “outed” and losing anonymity diminished her ability to succeed in monitoring her peers. At the same time, she could not educate her peers and promote hand hygiene on their unit, while remaining an anonymous HH champion.

In addition, some infection control teams whose HH champions had dual responsibilities suggested it would be more effective to separate the educational component from surveillance.

*And what we needed were people that didn’t work on the units, so we needed respiratory therapists. We needed EMS (Environmental Management Service). They were great. People passing trays, the nursing supervisors ( … ) you’re always expected to be everywhere all the time. Those people are your secret observers. ‘Cause nobody says to them, “What are you doin’ here?” You know? And then if we had light duty people, we would give them a good cover story of like why they were there, what they were doing, who they were helping. AND if anybody’s cover got blown, they were immediately removed from being an observer. So that’s how we got TRULY secret observations. [infection preventionist, site 9, T2]*

## Discussion

HH champions are used as an implementation strategy across many hospitals in the VHA to help improve hand hygiene compliance. Our ethnographic study examined how HH champions are being employed in “everyday” healthcare settings [20]. Our findings indicate many hand hygiene programs have merged at least two different implementation strategies into the role of the HH champion: (1) audit and feedback (hand hygiene surveillance) and (2) champions (promotion, coaching, and education). Analyses indicated that this description of the HH champion role tends to be contradictory. While frontline staff are accessible on the units to conduct surveillance and real-time coaching when they see non-compliant HCWs, their surveillance data are considered inaccurate because HCWs are more compliant when they see the HH champion. Participants both explicitly and implicitly referenced the Hawthorne effect in these instances [30]. In addition, we found the HH champions often feel pressure to capture and report the “good” hand hygiene of their peers on their unit. Barriers also include having to balance patient care and HH champion responsibilities at their facility. Other studies have demonstrated similar findings in which facilities struggled to implement clinical champions where different hierarchical levels did not have functional relationships [31–34]. On the other hand, we found important benefits to using HH champions (in the standard definition). Frontline staff in HH champion roles can help change the culture on their floor to improve hand hygiene compliance through the promotion of hand hygiene, informal coaching, and reminding their peers of proper hand hygiene behavior. In addition, although the accuracy of data is often questioned, HH champions as described by participants in this study supply an important patient safety metric.

Influencing change in providers’ behavior is difficult. Evidence suggests we need to “identify and prepare champions” to support the interventions or practice being implemented [9]. However, it is difficult to understand the role of champions, since a variety of terms have been used in healthcare-related implementation literature [35]. While the terms are different—“change agent” and “opinion leader”—they typically refer to the construct of the “champion” [35]. Miech and colleagues [35] identified over 26 characteristics of effective champions ranging from enthusiasm and energy to drive the implementation process to strong educator and presentation skills to having political acumen. With all of these skills making an effective champion, it is not without reason that applications of the implementation strategy in real-world healthcare settings have expanded to include additional skills and responsibilities such as audit and feedback. In addition, we conducted an ethnographic study to examine the real-world implementation of hand hygiene programs and found all 10 geographically dispersed sites used the term HH champion to describe a core component of their hand hygiene program. For this reason, we compared their utilization of the term to that used by implementation experts [9]. However, the barriers we found associated with using champions as an implementation strategy are similar to those found in recent work in the area of facilitation as an implementation strategy [36, 37]. The work in the area of facilitation points to a need to assess the context for the best-matched type of facilitation, ranging from task or goal-oriented to holistic, emancipatory approaches [38]. With all ten sites using the term champion in a similar way, it suggests little assessment and tailoring of implementation strategies to fit the context.

Although we focused on the term champions, surveillance was described as a key component of the HH champion’s role. In the taxonomy of implementation strategies, surveillance falls under audit and feedback, which has been defined as, “collect and summarize clinical performance data over a specified time period and give it to clinicians and administrators to monitor, evaluate, and modify provider behavior” [9, p. 8]. Audit and feedback have been used for decades to influence provider behavior. Research suggests that providing feedback to clinicians about their behavior on a recurring basis leads to important improvements in performance [39]. However, in a systematic review specific to hand hygiene, Gould and colleagues [4] found it difficult to draw conclusions on whether or not audit and feedback interventions could be sustained, largely due to the Hawthorne effect influence on performance behavior. Our own research building off of the same ethnographic study points to multiple barriers to the use of audit and feedback as a strategy for hand hygiene compliance, particularly when using direct observation as the surveillance method [40]. On the other hand, studies have also shown success with designated HCWs providing immediate hand hygiene feedback [15–17]. Further, Patel and colleagues (2016) echoed some of the same barriers in their study of HH champions, including time constraints to performing their daily responsibilities and champion duties, staff shortages, and turnover of the HH champion [17]. Patel and colleagues’ study use of HH champions was within the definitional boundaries described by implementation scientists [9, 34]. We are aware of no other hand hygiene compliance study that has used champion in this way. At the same time, many research studies have used audit and feedback, conducted both covertly and openly, as an implementation strategy to improve hand hygiene. Schweizer et al. [41] completed a systematic review and meta-analysis of hand hygiene improvement trials. Combined results from 39 quasi-experimental and six randomized trials indicated that bundles that included audit and feedback were associated with an 82% increase in hand hygiene [40]. Gould and colleagues [4] found that all 26 studies that met the criteria for inclusion reported some improvement in hand hygiene compliance; however, they were unable to draw conclusions about which interventions or combination of interventions led to clinically important improvements in compliance.

Being tasked with contradictory roles of both “championing” and surveillance audits can be problematic. In real-world settings, fidelity to implementation strategies is often not monitored or measured. As the field of implementation science matures, it is critical to examine how implementation strategies are being used and defined in real-world settings. In addition, implementation science may need to provide guidance for these instances when multiple implementation strategies are being combined or strategies expand beyond the definitional boundaries employed in the implementation science literature. While utilizing champions is an effective approach to eliciting change [31, 32, 42, 43], what guidance can implementation science offer to facilities using HH champions as a strategy in the real world? In addition, other implementation strategies should be explored to promote hand hygiene compliance.

Qualitative research in infection prevention has increased significantly over the past decade, including in the area of hand hygiene [44–46]. The number of studies using ethnographic methods to examine infection prevention interventions has increased since Dixon-Woods and colleagues examined the Michigan Keystone Project [47] and its attempted replication in England [19, 48]. One of the strengths of ethnographic and qualitative research is its inductive nature in which engagement with the key individuals and the context can lead to new or more in-depth knowledge and understanding of an area. The ethnographic work being done in infection control and reviewed by Knobloch and colleagues provides evidence of the diversity of deep knowledge that has emerged and strengthened the field from the unintended consequence of mandatory reporting [49] to social and organizational issues that impact hospital-acquired infection rates such as staff shortages and overcrowding [50]. However, few ethnographic studies have focused specifically on hand hygiene [51]. In a very creative and powerful use of ethnographic methods, Hor and colleagues [51] used the method of video reflexive ethnography as a quality improvement intervention in addition to semi-structured interviews and field observations. More broadly, Smiddy and colleagues [46] conducted a systematic qualitative literature review of hand hygiene compliance and found themes could be categorized into two factors: motivational and perceptions of the work environment. These two categories map on to areas often cited as the strengths of ethnographic or qualitative work: stakeholder meaning and structural context [52]. Gilbert and Kerridge [53] is one example in the field of hand hygiene that richly illuminates the well-documented pattern of physicians being among the least compliant hand washers among healthcare workers. Their in-depth qualitative work in a large, tertiary Australia hospital points to deep cultural, political, and ethical meaning and belief systems that may be contributing to seemingly incongruent pattern of lower compliance, while studies in developing countries point to broader structural and cultural influences impacting hand hygiene compliance among staff within the healthcare settings [54, 55]. In our own work, we conducted an ethnographic study to examine the everyday practices of hand hygiene *programs* at 10 different hospitals and found a pattern of a commonly employed implementation strategy: clinical champions. This finding not only has potentially important implications for the hand hygiene field, but also the field of implementation science. This sample of ethnographic and qualitative work focused on hand hygiene highlights points to the need for additional work in this area to examine additional deeply rooted cultural belief systems that may be contributing to compliance or methods of surveillance, as well as the structural and contextual barriers to compliance.

This study is not without limitations. First, our study is restricted to individuals within the VA Healthcare System. Other healthcare systems may use HH champions differently. Second, our sample was restricted to infection control teams and other individuals involved with hand hygiene and frontline staff available on days the study team visited each facility. Further, phone interviews were limited to only key staff involved with hand hygiene who agreed to participate, and therefore may not be entirely representative of all VA employees. Findings are based on self-report. Although we did observe hand hygiene program practices among some infection preventionists, we did not observe unit level HH champions as this thematic content emerged from the data and was not a specific element of original data collection. Further ethnographic study of HH champions—including observations of their activities—could serve as the next steps to elucidating the real-world definition and application of the implementation strategy in healthcare contexts. Finally, having an interdisciplinary research team provided the opportunity to challenge the biases we each brought to data collection and analyses. We tracked these conversations in team meetings and in the memo function of MAXQDA software. However, as with all reflexive processes, we may have missed biases that contributed to the interpretation of the results.

## Conclusions

The primary findings of this ethnographic study are twofold. First, it demonstrates how the definition of an implementation strategy (i.e., champion) can move beyond the original definition and encompass elements of additional implementations strategies (e.g., education and training, audit and feedback). This poses an important question to the field of implementation science: how do we understand the effectiveness of strategies when they are combined in ways that have not been evaluated? Secondly, for the field of infection prevention, healthcare systems should consider whether narrowly defining the role of the HH champion as a dedicated individual whose mission is to support hand hygiene and overcome resistance within an organization—and differentiate it from the role of a “compliance auditor”—could be more impactful in increasing hand hygiene compliance. Returning to the traditional application of the implementation strategy may lead to overall improvements in hand hygiene and reduction of the transmission of healthcare-acquired infections.

## Data Availability

The datasets generated and/or analyzed during the current study are not publicly available due to participant privacy but are available from the corresponding author on reasonable request.

## Abbreviations

EMS: Environmental Management Service
HAIs: Healthcare acquired infections
HCW: Healthcare worker
HH: Hand hygiene
HSR&D: Health Services Research and Development
MDRO: Multi-drug-resistant organisms
VA: Veteran’s Affairs
VAHCSs: Veterans Affairs Healthcare Systems
VHA: Veterans Health Administration
WHO: World Health Organization
